# Supplementary material for: Self-encapsulated ionic fibers based on stress-induced adaptive phase transition for non-contact depth-of-field camouflage sensing
Source: Nat Commun. 2024 Jan 22;15:663. doi: 10.1038/s41467-024-44848-5 (PMC10803323; doi:10.1038/s41467-024-44848-5)
Supplement: Supplementary file 1 — Supplementary Information [file 41467_2024_44848_MOESM1_ESM.pdf]

Supplementary Information

Self-encapsulated Ionic Fibers Based on Stress-induced Adaptive Phase Transition for Non-contact Depth-of-Field Camouflage Sensing

Ying Liu[1,2,†], Chan Wang[1,2,†], Zhuo Liu[1,3,†], Xuecheng Qu[1,2], Yangsong Gai[1], Jiangtao Xue[1,4], Shengyu Chao[1,2], Jing Huang[1,2], Yuxiang Wu[1,5], Yusheng Li[1,6], Dan Luo[1,2*], Zhou Li[1,2*]

[1]Beijing Institute of Nanoenergy and Nanosystems, Chinese Academy of Sciences, Beijing 101400, China.

[2]School of Nanoscience and Engineering, University of Chinese Academy of Sciences, Beijing 100049, China.

[3]Key Laboratory of Biomechanics and Mechanobiology, Ministry of Education, Beijing Advanced Innovation Center for Biomedical Engineering, School of Engineering Medicine, Beihang University, Beijing, 100191, China.

[4]School of Life Science, Institute of Engineering Medicine, Beijing Institute of Technology, Beijing 100081, China

[5]Department of Health and Kinesiology, School of Physical Education, Jianghan University, Wuhan 430056, China

[6]National Clinical Research Center for Geriatric Disorders, Xiangya Hospital, Central South University, Changsha 410008, China

*Correspondence authors. luodan@binn.cas.cn (Dan Luo), zli@binn.cas.cn (Zhou Li)

†These authors contributed equally to this work.

Supplementary Figures

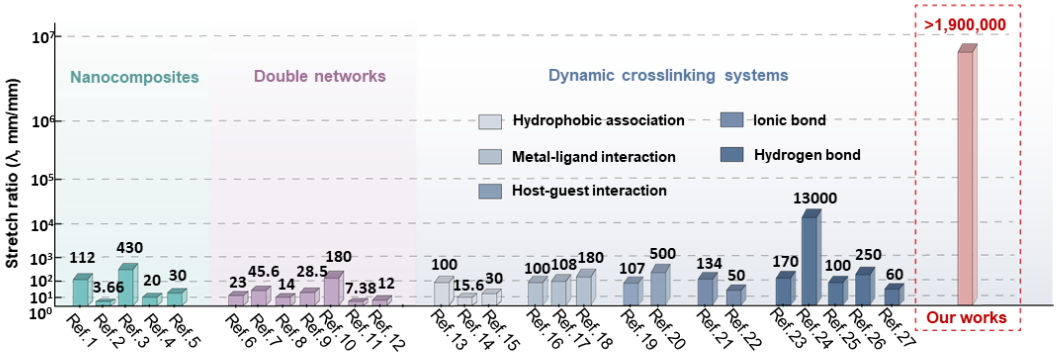

Supplementary Fig. 1 A comparison between us-IHs and previously reported stretchable polymeric materials in terms of stretchability. The details are summarized in Supplementary Table 1.

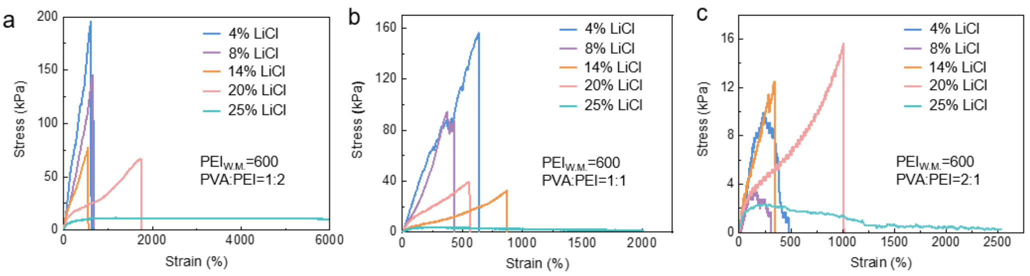

Supplementary Fig. 2 The stress-strain curves of hydrogels with different LiCl contents (4%, 8%, 14%, 20% and 25%): (a) hydrogel with a 1:2 mass ratio of PVA to PEI, (b) hydrogel with a 1:1 mass ratio of PVA to PEI, (c) hydrogel with a 2:1 mass ratio of PVA to PEI, and the molecular weight of PEI used in all hydrogels was 600 Da. When the addition amount of LiCl was 25%, all samples exhibited the greatest stretchability.

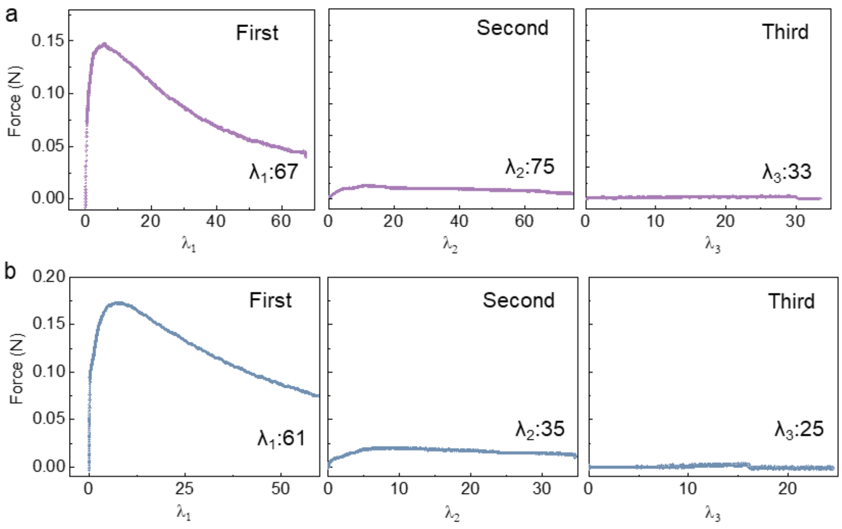

Supplementary Fig. 3 The stress-strain curves of (a) $_{1}$PVA-$_{2}$PEI$_{600}$-LiCl$_{25}$, (b) $_{1}$PVA-$_{1}$PEI$_{600}$-LiCl$_{25}$.

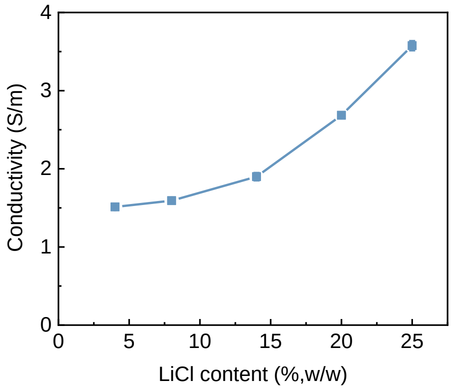

Supplementary Fig. 4 The conductivity of hydrogels with different LiCl doped. The conductivity of hydrogels gradually increased with the addition of LiCl.

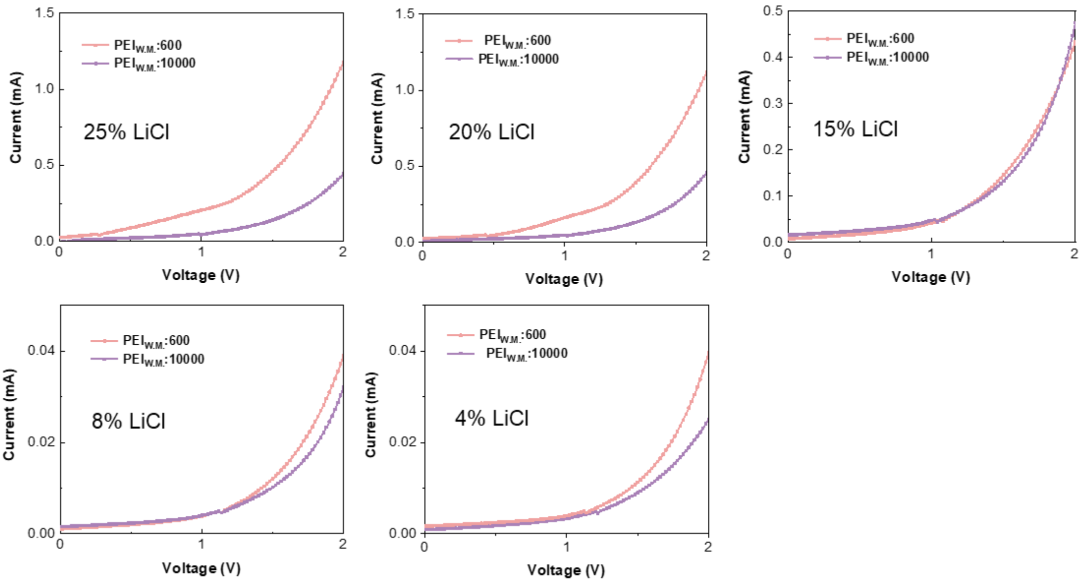

Supplementary Fig. 5 The conductivity of hydrogel with different PEI molecular weight (600 and 10000) were tested by Semiconductor Analysis System.

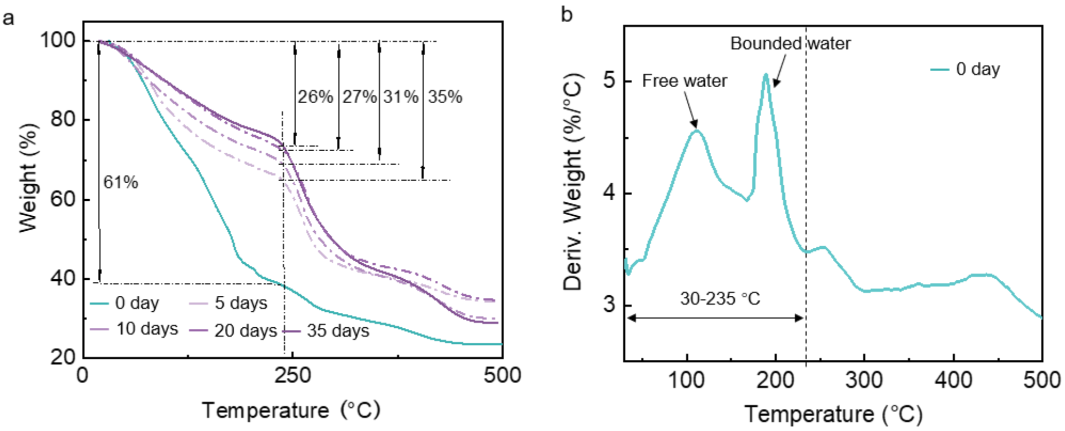

Supplementary Fig. 6 (a) The representative TGA curves of fibers with different self-encapsulation times. (b) The DTG cures of fibers at 0 day.

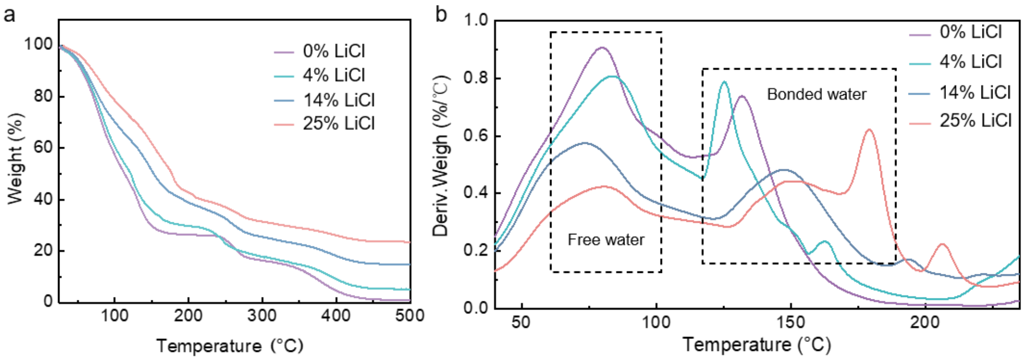

Supplementary Fig. 7 The TGA (a) and DTG (b) curves for hydrogel with different LiCl doped.

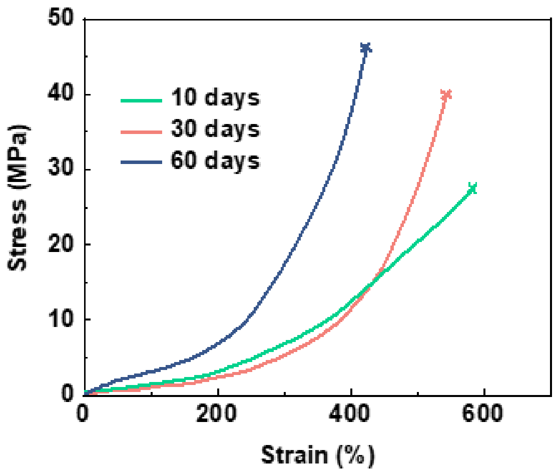

Supplementary Fig. 8 The stress-strain curves of se-HICFs with different dehydration time. The mechanical strength of se-HICFs gradually increased during the self-encapsulation process.

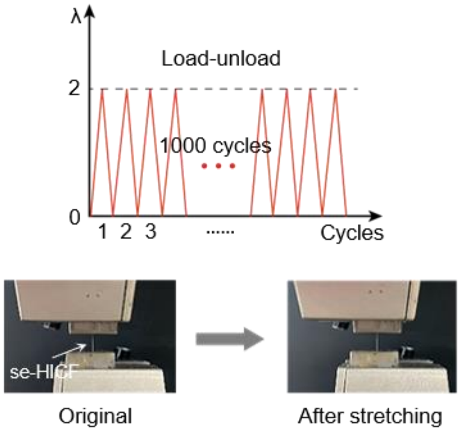

Supplementary Fig. 9 The tensile fatigue stability of se-HICF. In the stretching mode, se-HICF could recover to its original length after 1000 loading-unloading cycles, showing great robustness and fatigue resistance.

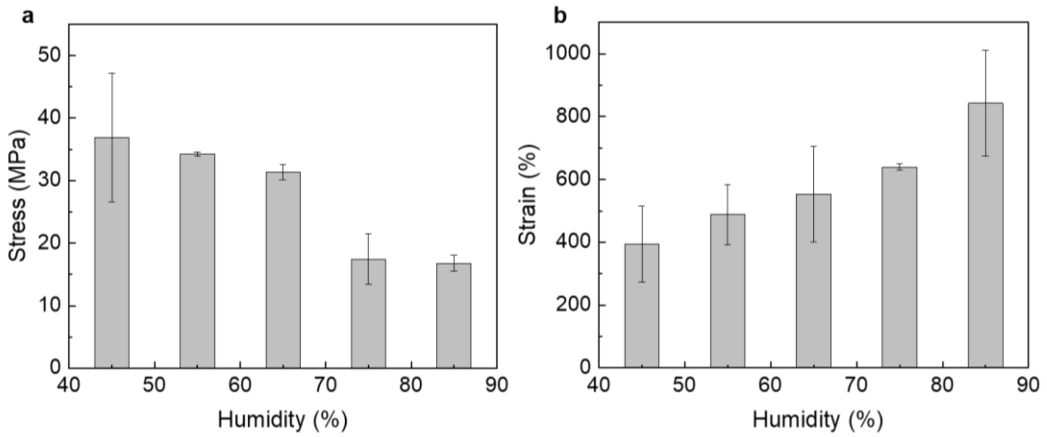

Supplementary Fig. 10 The stress (a) and strain (b) of the se-HICFs under different humidity on the test of stress-strain curve. The error bar for each data point in **a**, **b** is standard deviation calculated based on 3 parallel measurements.

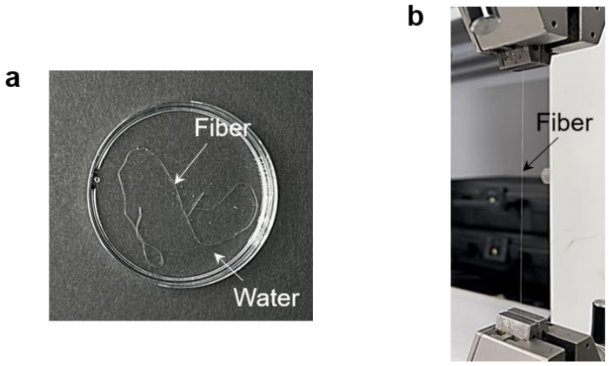

Supplementary Fig. 11 (a) The photography of fiber immersing in the water after 60 min.(b) The se-HICFs during the stretching test.

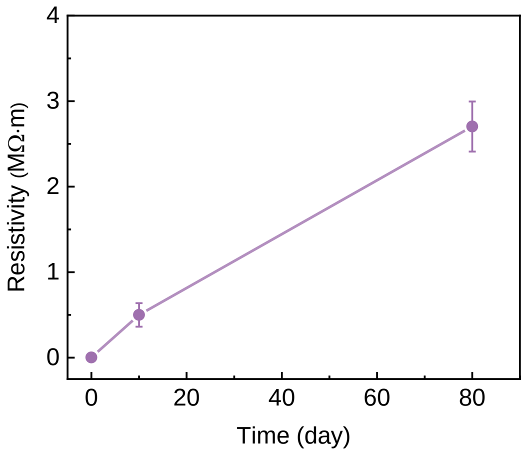

Supplementary Fig. 12 The resistivity of us-IHs with different time. It could be the insulated layer after the evaporation of water. The error bar for each data point is standard deviation calculated based on 3 parallel measurements.

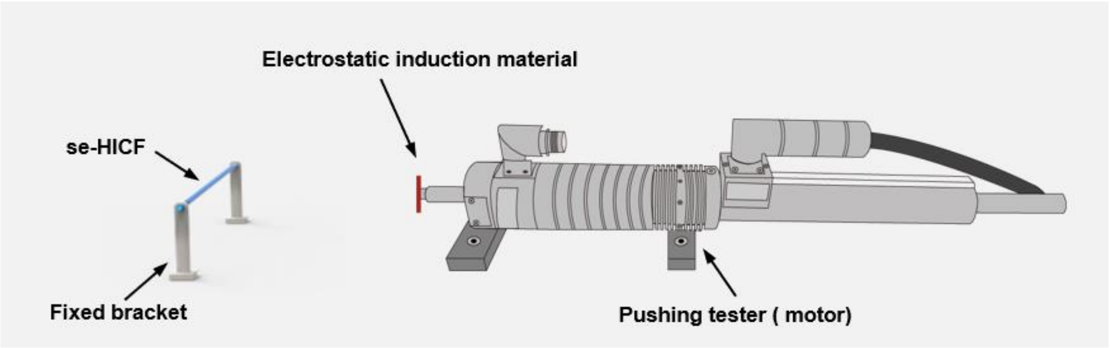

Supplementary Fig. 13 The schematic diagram of electrostatic induction sensing test. Use a pushing tester to control movement speed and gap distance.

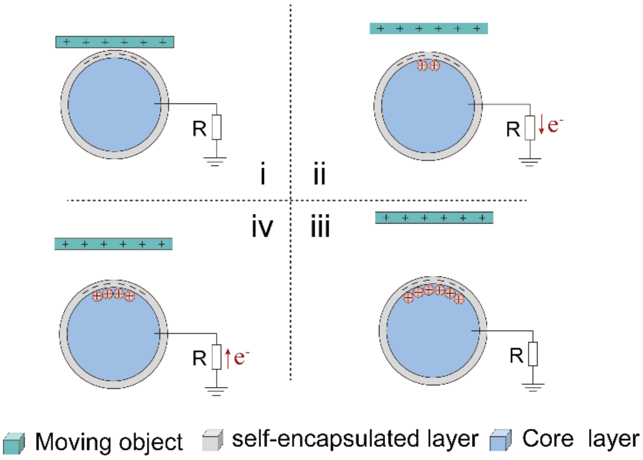

Supplementary Fig. 14 The sensing mechanism of se-HICF, as a single-electrode triboelectric nanogenerator, under a contact model of a moving object with se-HICF. Once a positively charged moving object contacted with se-HICF, the triboelectric effect occurred at the contact interface, and the self-encapsulated sheath layer generated an opposite charge to that of the moving object. When the object moved away, the negative charges on the surface of the self-encapsulated layer induced an outflow of electrons from the core layer of se-HICF to balance them, producing an output electrical signal as the electrons passed through the external circuit. Conversely, when the moving object approached se-HICF, the entire process was reversed, and the electrons flowed into the core layer of se-HICF to produce a reverse output signal. Therefore, the movement of the target material can be identified using either the open-circuit voltage or the produced alternating current.

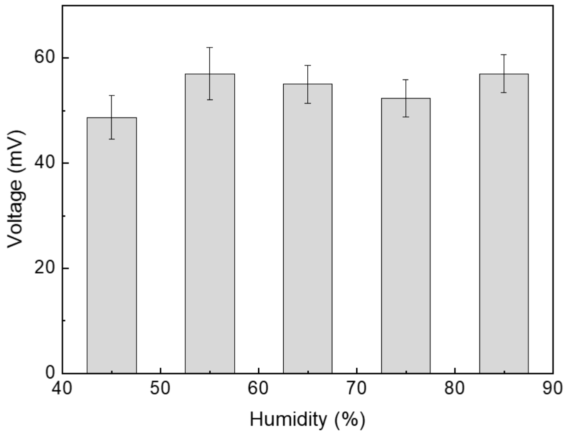

Supplementary Fig. 15 The stability of electrostatic induction ability for se-HICFs under different humidity conditions. The error bar for each data point is standard deviation calculated based on 3 parallel measurements.

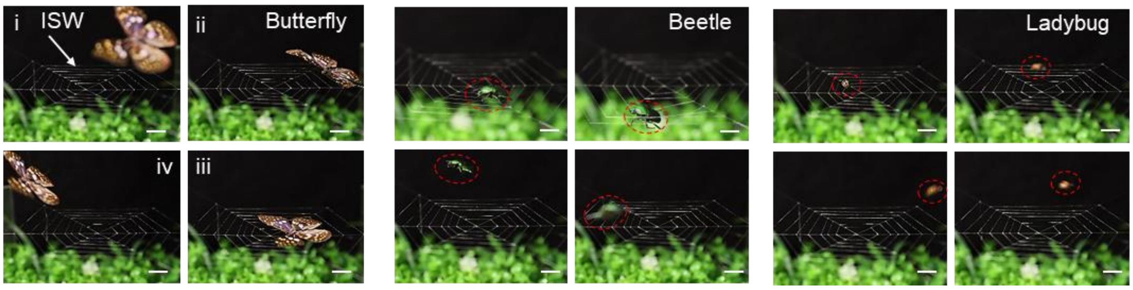

Supplementary Fig. 16 Photographs of different insects (butterfly, beetle and ladybug) fly on the ISW. Scale bar: 1.5 cm.

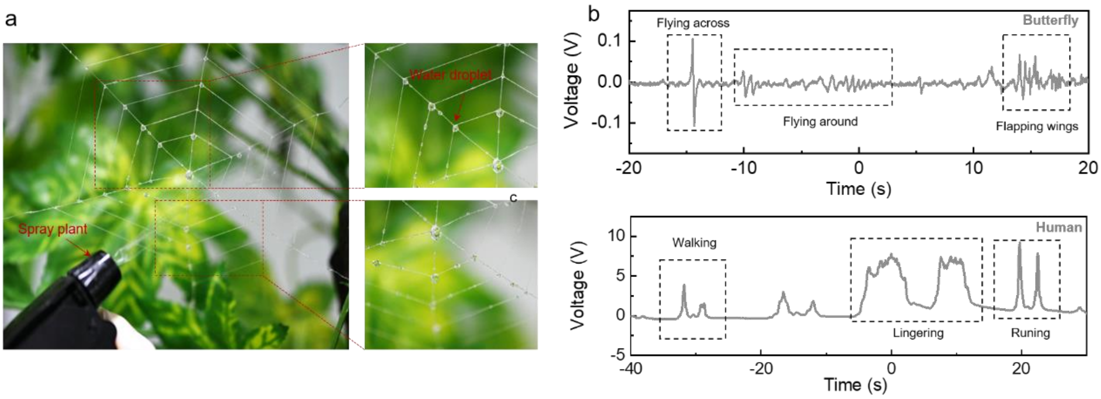

Supplementary Fig. 17 (a) Simulation of a rainy day. The V_{oc} of ISW sensing the moving (b) butterfly and (c) human.

Table S1. The comparison of us-IHs and other related works.

Ref.	Stretching strategy	Composites	Stretchability (λ)	Strength (kPa)	Stretching rate (mm/min)	Application
This work	Hydrogen bond	PVA/PEI/LiCl	>1,900,000	36	30	Flexible sensor
1	nanocomposites	PAM/cement	112	400	-	-
2		PVA/CFP	3.66	899	16	Strain sensor
3		PAM/CNS	430	121	50	-
4		Fe_3O_4NPs/PAA	20	-	100	Strain sensor
5		Alginate/AAm/AuNR	30	3.64	100	-
6	Double network (DN)	Alginate/PAM	23	156	-	-
7		KOH/guanosine/PAAm	45.6	460	100	electrolyte
8		Chitosan/polysulfobetaine	14	80	100	Electric skin
9		Gelatin/PAM/PEDOT:PSS	28.5	298	50	Stain sensor (TENG)
10		PAAM/PVP	180	179±28.1	100	supercapacitors
11		PAM/CNF/LiCl	7.48	-	50	electrolyte
12		PNN/chitosan	12	235	10	ACEL device
13	Hydrophobic association	UPyHCBA	>100	3.92±0.87	15, 60, 240, 960	-
14		PMAA/PDMAEMA	11.5-15.6	61-103.4	5	-

15		CNT_S/HAPAAm	30	192	50	Pressure sensor
16	Metal coordination	Fe-Hpdca-PDMS	>100	~50	2	-
17		Q-PEI/PAA	108	3000	40	-
18		CSH/PPG/Zn	180	320	100	-
19	Host-guest interaction	CB/PAM	107	1800	-	-
20		Azo-PAM/α-CDP/LiCl	500	-	-	Supercapacitor
21	Ionic bond	PI	134	7000	30	-
22		PVDF-co-HFP	50	100	5	-
23	Hydrogen bond	SPMs	170	910	100	Electrode
24		PB-imine	13000	-	10	-
25		CMA/PVA	>100	-	60	-
26		SF/TA/rGO	250	191±17	100	-
27		PDA/PAM	60	~300	-	Electrode

—: Not mentioned

Table S2. The components of the ionic hydrogel.

Sample Number	Sample Name	PVA (g)	PEI (g)	PEI (W.M.)	DI water (mL)	LiCl (g)	LiCl content (W/W)	Strain (%)	Stress (kPa)
1	$_2$PVA-$_1$PEI$_{600}$-LiCl$_4$	2	1	600	9	0.5	4	486	10
2	$_2$PVA-$_1$PEI$_{600}$-LiCl$_8$	2	1	600	9	1	8	366	4
3	$_2$PVA-$_1$PEI$_{600}$-LiCl$_{14}$	2	1	600	9	2	14	349	13
4	$_2$PVA-$_1$PEI$_{600}$-LiCl$_{20}$	2	1	600	9	3	20	1020	16
5	$_2$PVA-$_1$PEI$_{600}$-LiCl$_{25}$	2	1	600	9	4	25	---	3
6	$_1$PVA-$_1$PEI$_{600}$-LiCl$_4$	1.5	1.5	600	9	0.5	4	640	94
7	$_1$PVA-$_1$PEI$_{600}$-LiCl$_8$	1.5	1.5	600	9	1	8	435	156
8	$_1$PVA-$_1$PEI$_{600}$-LiCl$_{14}$	1.5	1.5	600	9	2	14	564	40
9	$_1$PVA-$_1$PEI$_{600}$-LiCl$_{20}$	1.5	1.5	600	9	3	20	877	33
10	$_1$PVA-$_1$PEI$_{600}$-LiCl$_{25}$	1.5	1.5	600	9	4	25	---	4
11	$_1$PVA-$_2$PEI$_{600}$-LiCl$_4$	1	2	600	9	0.5	4	903	250
12	$_1$PVA-$_2$PEI$_{600}$-LiCl$_8$	1	2	600	9	1	8	1197	290
13	$_1$PVA-$_2$PEI$_{600}$-LiCl$_{14}$	1	2	600	9	2	14	874	54
14	$_2$PVA-$_1$PEI$_{600}$-LiCl$_{20}$	1	2	600	9	3	20	1755	67
15	$_2$PVA-$_1$PEI$_{600}$-LiCl$_{25}$	1	2	600	9	4	25	---	11
16	$_2$PVA-$_1$PEI$_{10000}$-LiCl$_4$	2	1	10000	9	0.5	4	2091	1636
17	$_2$PVA-$_1$PEI$_{10000}$-LiCl$_8$	2	1	10000	9	1	8	3164	1362
18	$_2$PVA-$_1$PEI$_{10000}$-LiCl$_{14}$	2	1	10000	9	2	14	3864	963
19	$_2$PVA-$_1$PEI$_{10000}$-LiCl$_{20}$	2	1	10000	9	3	20	4467	657
20	$_2$PVA-$_1$PEI$_{10000}$-LiCl$_{25}$	2	1	10000	9	4	25	6919	332

---: Not fractured

Table S3. The comparison of se- HICFs and other conductive hydrogel fibers.

Ref.	Materials	Strength	Stretchability
This work	PVA-PEI-LiCl	27, 40, 46 MPa	583%, 542%, 422%
1[26]	Hydrogel	5.6 MPa	1200%
2[35]	PVDF	2-4 kPa	8%
3[36]	PVC gel	300 kPa	53%
4[37]	CNT	17.8 MPa	1.3%
5[38]	Silk	12.5 MPa	47%
6[39]	Liquid metal	2.16 MPa	1170%
7[40]	GO/G	100 MPa	5%

Supplementary References

1. Liang, R., Li, Z., Weng, L.-T., Zhang, L. & Sun, G. Recoverable hydrogel with high stretchability and toughness achieved by low-temperature hydration of Portland cement. *Mater. Chem. Front.* **2**, 2076-2080 (2018).
2. Cheng, B. *et al.* Highly Stretchable and Compressible Carbon Nanofiber–Polymer Hydrogel Strain Sensor for Human Motion Detection. *Macromol. Mater. Eng.* **305**, (2020).
3. Sun, G., Li, Z., Liang, R., Weng, L. T. & Zhang, L. Super stretchable hydrogel achieved by non-aggregated spherulites with diameters <5 nm. *Nat. Commun.* **7**, 12095, (2016).
4. Zhang, L. M. et al. Self-Healing, Adhesive, and Highly Stretchable Ionogel as a Strain Sensor for Extremely Large Deformation. *Small* **15**, e1804651 (2019).
5. Turner, J. G., Og, J. H. & Murphy, C. J. Gold nanorod impact on mechanical properties of stretchable hydrogels. *Soft Matter* **16**, 6582-6590 (2020).
6. Sun, J. Y. et al. Highly stretchable and tough hydrogels. *Nature* **489**, 133-136, (2012).
7. Gu, C. et al. Small molecule-based supramolecular-polymer double-network hydrogel electrolytes for ultra-stretchable and waterproof Zn–air batteries working from −50 to 100 °C. *Energy Environ. Sci.* **14**, 4451-4462, (2021).
8. Zhang, L. M. *et al.* Self-Healing, Adhesive, and Highly Stretchable Ionogel as a Strain Sensor for Extremely Large Deformation. *Small* **15**, e1804651 (2019).
9. Sun, H. et al. Ultra-Stretchable, durable and conductive hydrogel with hybrid double network as high performance strain sensor and stretchable triboelectric nanogenerator. *Nano Energy* **76**, 105035 (2020).
10. Zhang, H., Niu, W. & Zhang, S. Extremely stretchable, sticky and conductive double-network ionic hydrogel for ultra-stretchable and compressible supercapacitors. *Chem. Eng. J.* **387**, 124105 (2020).
11. Ge, W., Cao, S., Yang, Y., Rojas, O. J. & Wang, X. Nanocellulose/LiCl systems enable conductive and stretchable electrolyte hydrogels with tolerance to dehydration and extreme cold conditions. *Chem. Eng. J.* **408**, 127306 (2021).
12. Dinh Xuan, H. *et al.* Super Stretchable and Durable Electroluminescent Devices Based on Double-Network Ionogels. *Adv. Mater.* **33**, e2008849, (2021).
13. Jeon, I., Cui, J., Illeperuma, W. R., Aizenberg, J. & Vlassak, J. J. Extremely Stretchable and Fast Self-Healing Hydrogels. *Adv Mater* **28**, 4678-4683 (2016).

14. Heng Chen, B. H., Penghui Ge and Shaojun Chen. Highly stretchable, self-healing, and 3D printing prefabricatable hydrophobic association hydrogels with the assistance of electrostatic interaction. *Polym. Chem.* **11**, 4741–4748 (2020).
15. Qin, Z. *et al.* Carbon Nanotubes/Hydrophobically Associated Hydrogels as Ultrastretchable, Highly Sensitive, Stable Strain, and Pressure Sensors. *ACS Appl. Mater. Interfaces* **12**, 4944-4953 (2020).
16. Li, C. H. *et al.* A highly stretchable autonomous self-healing elastomer. *Nat. Chem.* **8**, 618-624 (2016).
17. Das Mahapatra, R., Imani, K. B. C. & Yoon, J. Integration of Macro-Cross-Linker and Metal Coordination: A Super Stretchable Hydrogel with High Toughness. *ACS Appl. Mater. Interfaces* **12**, 40786-40793 (2020).
18. Xu, J. *et al.* Extremely Stretchable, Self-Healable Elastomers with Tunable Mechanical Properties: Synthesis and Applications. *Chem. Mater.* **30**, 6026-6039, (2018).
19. Liu, J. *et al.* Tough Supramolecular Polymer Networks with Extreme Stretchability and Fast Room-Temperature Self-Healing. *Adv. Mater.* **29**, 1605325 (2017).
20. Kim, J. *et al.* Stretchable, self-healable, and photodegradable supercapacitor based on a polyelectrolyte crosslinked via dynamic host-guest interaction. *Chem. Eng. J.* **422**, (2021).
21. Miwa, Y., Kurachi, J., Kohbara, Y. & Kutsumizu, S. Dynamic ionic crosslinks enable high strength and ultrastretchability in a single elastomer. *Commun. Chem.* **1**, 1-8 (2018).
22. Cao, Y. *et al.* A Transparent, Self-Healing, Highly Stretchable Ionic Conductor. *Adv. Mater.* **29**, 1605099 (2017).
23. Yan, X. *et al.* Quadruple H-Bonding Cross-Linked Supramolecular Polymeric Materials as Substrates for Stretchable, Antitearing, and Self-Healable Thin Film Electrodes. *J. Am. Chem. Soc.* **140**, 5280-5289 (2018).
24. Zhang, H., Niu, W. & Zhang, S. Extremely stretchable, sticky and conductive double-network ionic hydrogel for ultra-stretchable and compressible supercapacitors. *Chem. Eng. J.* **387**, (2020).
25. Chaudhary, J. P. *et al.* Carboxymethylagarose-based multifunctional hydrogel with super stretchable, self-healable having film and fiber forming properties. *Arab. J. Chem.* **13**, 1661-1668 (2020).

26. Kadumudi, F. B. *et al.* The Manufacture of Unbreakable Bionics via Multifunctional and Self-Healing Silk-Graphene Hydrogels. *Adv. Mater.***33**, e2100047, (2021).
27. Long, Y. *et al.* A flexible triboelectric nanogenerator based on a super-stretchable and self-healable hydrogel as the electrode. *Nanoscale* **12**, 12753-12759 (2020).
