# Supplementary material for: Self-encapsulated ionic fibers based on stress-induced adaptive phase transition for non-contact depth-of-field camouflage sensing
Source: Nat Commun. 2024 Jan 22;15:663. doi: 10.1038/s41467-024-44848-5 (PMC10803323; doi:10.1038/s41467-024-44848-5)
Supplement: Supplementary file 3 — Description of Additional Supplementary Files [file 41467_2024_44848_MOESM3_ESM.pdf]

Description of Additional Supplementary Files

Supplementary Movies 1 : The mechanical property of hydrogel fibers without selfencapsulation.

Supplementary Movies 2 : Puncture-resistant ability of us-IHs film.

Supplementary Movies 3 : Multistage stretching of us-IHs into ionic fibers.

Supplementary Movies 4 : Dip-drawing of us-IHs into ionic fibers.

Supplementary Movies 5 : Drawing with different needles.

Supplementary Movies 6 : The stretchability of ionic fibers.
